# Mass concentration in a nonlocal model of clonal selection

**DOI:** 10.1007/s00285-016-0979-3

**Published:** 2016-03-03

**Authors:** J.-E. Busse, P. Gwiazda, A. Marciniak-Czochra

**Affiliations:** 1 Institute of Applied Mathematics, BIOQUANT, University of Heidelberg, Im Neuenheimer Feld 294, 69120 Heidelberg, Germany; 2 Institute of Applied Mathematics and Mechanics, University of Warsaw, ul. Banacha 2, 02-097 Warsaw, Poland; 3 Institute of Mathematics, Polish Academy of Science, Śniadeckich 8, 00-656 Warszawa, Poland; 4 Interdisciplinary Center of Scientific Computing (IWR), University of Heidelberg, Im Neuenheimer Feld 205, 69120 Heidelberg, Germany; 5 Bioquant, University of Heidelberg, Im Neuenheimer Feld 205, 69120 Heidelberg, Germany

**Keywords:** Integro-differential equations, Mass concentration, Lyapunov function, Selection process, Clonal evolution, Cell differentiation model, Bounded Lipschitz distance, 92D25

## Abstract

Self-renewal is a constitutive property of stem cells. Testing the cancer stem cell hypothesis requires investigation of the impact of self-renewal on cancer expansion. To better understand this impact, we propose a mathematical model describing the dynamics of a continuum of cell clones structured by the self-renewal potential. The model is an extension of the finite multi-compartment models of interactions between normal and cancer cells in acute leukemias. It takes a form of a system of integro-differential equations with a nonlinear and nonlocal coupling which describes regulatory feedback loops of cell proliferation and differentiation. We show that this coupling leads to mass concentration in points corresponding to the maxima of the self-renewal potential and the solutions of the model tend asymptotically to Dirac measures multiplied by positive constants. Furthermore, using a Lyapunov function constructed for the finite dimensional counterpart of the model, we prove that the total mass of the solution converges to a globally stable equilibrium. Additionally, we show stability of the model in the space of positive Radon measures equipped with the flat metric (bounded Lipschitz distance). Analytical results are illustrated by numerical simulations.

## Introduction

This paper is devoted to the analysis of a structured population model describing clonal evolution of acute leukemias. Leukemia is a disease of the blood production system leading to an extensive expansion of malignant cells that are non-functional and cause an impairment of blood regeneration. Recent experimental evidence indicates that cancer cell populations are composed of multiple clones consisting of genetically identical cells (Ding et al. 2012) and maintained by cells with stem-like properties (Bonnet and Dick 1997; Hope et al. 2004). Many authors have provided evidence for heterogeneity of leukemic stem cells (LSC) attempting to identify their characteristics; for review see Lutz et al. (2012). Heterogeneity is further supported by the results of gene sequencing studies (Ding et al. 2012; Ley et al. 2008). However, it was shown in these studies that a limited number of clones contribute to the total leukemic cell mass. At most 4 contributing clones were detected in the case of acute myeloid leukemia (AML) and at most 10 in the case of acute lymphoblastic leukemia (ALL) (Ding et al. 2012; Lutz et al. 2012). Moreover, in most cases of ALL, the clones dominating the relapse have already been present at the diagnosis but undetectable by the routine methods (Van Delft et al. 2011; Choi et al. 2007; Lutz et al. 2013). Due to a quiescence, a very slow cycling or other intrinsic mechanisms (Lutz et al. 2013; Choi et al. 2007), these clones may survive chemotherapy and eventually expand (Lutz et al. 2013; Choi et al. 2007). This implies that the main mechanism of relapse in ALL might be selection of existing clones and not acquisition of therapy-specific mutations (Choi et al. 2007). Similar mechanisms have been described in AML (Ding et al. 2012; Jan and Majeti 2013). Based on these findings the evolution of malignant cells can be interpreted as a selection process for properties that enable cells to survive the treatment and to expand efficiently. The mechanisms of the underlying process and its impacts on the disease dynamics and on the response of cancer cells to chemotherapy are not understood. Gene sequencing studies allow deciphering the genetic relations among different clones; nevertheless the impact of many detected mutations on cell behaviour remains unclear (Ding et al. 2012). The multifactorial nature of the underlying processes severely limits the intuitive interpretation of the experimental data.

To investigate the impact of cell properties on the multi-clonal composition of leukemias and to elucidate the possible mechanisms of the clonal selection suggested by the experimental data, a multi-compartmental model was proposed and studied numerically in Stiehl et al. (2014). It assumes the form of the following system of ordinary differential equations,1with nonnegative initial data.

The model describes time dynamics of a healthy cell line, denoted by $c_j$, $j=1,2$ and of *n* clones of leukemic cells $l^i_j$, for $j=1,2,$ and $i=1,\ldots ,n$, at time *t*. Each population consists of two different cell types, proliferating and non-proliferating, denoted by $j=1$ and $j=2$, respectively. This two-compartment model is a simplification of the more realistic model with multiple differentiation stages; see Marciniak-Czochra et al. (2009), Stiehl et al. (2013) for an introduction to the model and its application to the healthy hematopoiesis; Getto et al. (2013), Nakata et al. (2011), Stiehl and Marciniak-Czochra (2011) for its analysis; and Doumic et al. (2011) for a continuous-structure extension. This model can be viewed as a structured population model with a discrete structure describing two differentiation stages and $n+1$ cell types.

Parameters $p^c>0$ and $p^{l^i}>0$ denote the proliferation rate of the healthy cells and the cells in the leukemic clone *i*, respectively, and $a^c$ and $a^{l^i}$ are the corresponding maximal fractions of self-renewal, which depend on the proportion of symmetric and asymmetric cell divisions in the respective population. More precisely, the self-renewal fractions $0<a^c<1$ and $0<a^{l^i}<1$ are the fractions of the progeny cells that remain in the compartment of proliferating cells. Consequently, $(1- a^{c})$ and $(1- a^{l^i})$ are fractions of the dividing cells that differentiate and become non-proliferating. By $d_2^c>0$ and $d_2^{l^i}>0$ we denote the clearance rate of the non-proliferating healthy cells and the cells in the *i*th leukemic clone, respectively.

The model is based on the assumption that leukemic clones and their normal counterparts respond to a hematopoietic feedback signalling and compete for signalling factors (cytokines). We assume that the feedback signal, *s*(*t*), decreases if the number of non-proliferating cells increases. Derivation of such nonlinear feedback loop was proposed in Marciniak-Czochra et al. (2009). It is based on a Tikhonov-type quasi-stationary approximation of dynamics of the extracellular signalling molecules, such as the G-CSF cytokine, which are secreted by specialised cells at a constant rate and degraded by a receptor-mediated endocytosis. Following the evidence from clinical trials that the mature granulocytes mediate clearance of G-CSF (Layton et al. 1989), we assume that dynamics of the signalling molecules depends on the number of non-proliferating cells. This assumption has been also supported by studies of receptor expression showing that the mature cells express significantly more receptors than the cells in bone marrow (Shinjo et al. 1997). Taking into account these observations, we obtain a model with a nonlinear coupling depending on the level of non-proliferating cells.

Numerical simulations of model (1) suggest that cells with a superior self-renewal potential, i.e. a maximum value of the parameter *a*, reflecting the probability that a daughter cell has the same properties and fate as its parent cell, have an advantage in comparison to their competitors, which leads to the expansion of this cell subpopulation (Stiehl et al. 2014). The phenomenon was shown analytically solely in the case of two competing populations, a healthy and a cancerous cell line (Stiehl and Marciniak-Czochra 2012).

**Fig. 1:**
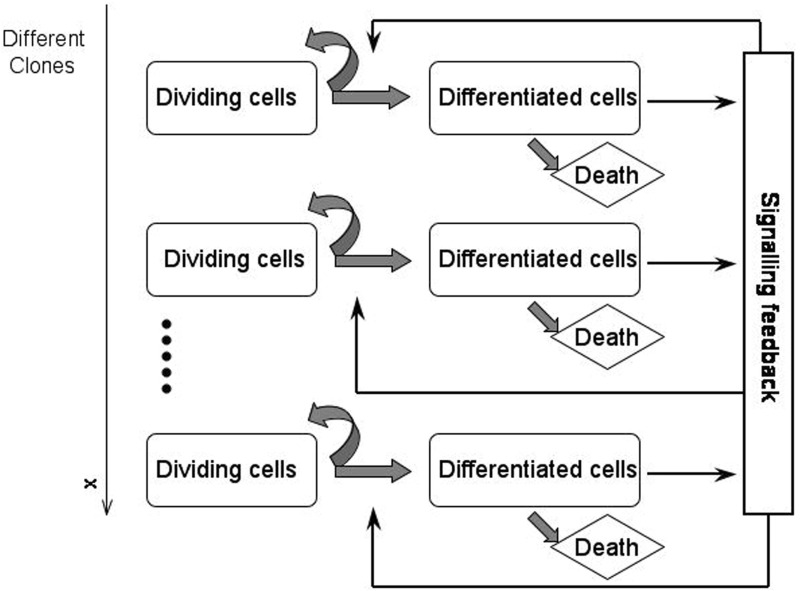
Schematic representation of model (2), consisting of two compartments corresponding to undifferentiated cells (dividing cells) and mature cells (differentiated cells). Undifferentiated cells (stem cells and early progenitors) divide symmetrically or asymmetrically. Accordingly, they produce cells of the same type (self-renewal) and mature cells (differentiation). Mature cells do not divide and they die after an exponentially distributed lifetime. The cells in each compartment are heterogenous. They are stratified by a structure variable *x* that represents the expression level of genes (yielding a phenotype and eg. influencing the self-renewal properties of the cells). Self-renewal and differentiation of cells are regulated by a cytokine feedback which, in turn, depends on the total count of differentiated cells

To elucidate further mechanisms of clonal selection, we propose an infinitely dimensional extension of the multi-compartment model (1). We introduce a continuous variable $x\in \varOmega$ that represents the expression level of genes (yielding a phenotype) influencing self-renewal properties of the cells. It leads to a system of integro-differential equations describing dynamics of a structured population with the continuum of cell clones and the two-compartment differentiation structure. Cells in Population 1 (dividing cells) proliferate and may self-renew or differentiate into Population 2 cells (differentiated cells). Population 2 cells do not proliferate and die after an exponentially distributed lifetime, as depicted in Fig. 1. Cells in both populations are stratified by a structure variable *x*. We assume that the self-renewal parameter depends on *x*, i.e. the parameter *a* becomes a function *a*(*x*). These assumptions lead to the model

2 $$\begin{aligned} \frac{\partial }{\partial t} u_1(t,x)= & {} \left( 2a(x)s(t)-1\right) pu_1(t,x), \nonumber \\ \frac{\partial }{\partial t} u_2(t,x)= & {} 2\left( 1-a (x)s(t) \right) pu_1(t,x) - d u_2(t,x), \nonumber \\ u_1(0,x)= & {} u_1^0(x), \nonumber \\ u_2(0,x)= & {} u_2^0(x). \end{aligned}$$

Assuming $s(t) = 1/(1+K\int \nolimits _{\varOmega } u_2 (t,x)\mathrm {d}x)$, we obtain a nonlocal and nonlinear coupling of the two equations.

Our approach is motivated by the theory of selection of the most fit variants in adaptive evolution. Cells with different mutational variants might have different growth properties allowing them to expand more efficiently. The phenomenon can be understood as an example of a process, which is closely related to Darwinian evolution. In our particular case, certain rare mutants may have positive growth rates and be selected in environments that otherwise result in extinction. In other words, cells with a fitness advantage expand and dominate dynamics of the population leading to extinction of the other cell clones. The model proposed belongs to the class of selection models exhibiting a mass concentration effect, similar to those presented in the books Perthame (2007) and Bürger (2000).

In the current work, we do not model mutation events. Instead, motivated by the experimental findings described earlier in Lutz et al. (2013), Choi et al. (2007), we aim to understand which aspects of the dynamics of leukemias can be explained by the selection alone. It is interesting, since the relapse caused by an expansion of a clone that could not be detected at diagnosis due to the limited sensitivity of detection methods, can be misinterpreted as a mutational event (Choi et al. 2007). A computational model of the AML with mutations was proposed in Stiehl et al. (2014). Following the biological evidence (Jan et al. 2012), it was assumed that new LSC clones were formed due to mutations occurring in LSCs or due to the influx from the so-called preleukemic cells at a rate modelled by a time inhomogeneous Poisson process. At each point of the Poisson process a new clone with random cell properties was added to the system. Simulations of that model demonstrate that leukemic cell properties at diagnosis and at relapse are comparable to the scenario without mutations. Introducing mutations to the continuous models is known to make asymptotic analysis more complicated, and therefore we do not consider this aspect in the current paper.

The mathematical angle of our study is analysis of the nonlocal effects and development of singularities in the solutions of the integro-differential equations. We show that the solutions of system (2) may tend to Dirac measures concentrated in points with the largest value of the self-renewal potential. Such dynamics can be interpreted in the terms of selection, which causes convergence of the heterogeneous initial data to a stationary solution with the mass localised on a set of measure zero. Convergence then holds in the weak$*$ topology of Radon measures. Considering the space of positive Radon measures with a suitable metric allows formulating the result on convergence of solutions to a stationary measure in the terms of the metric instead of the weak$*$ convergence of Radon measures. We apply the flat metric (bounded Lipschitz distance), which has proven to be useful in the analysis of a variety of transport equations models, for example to study Lipschitz dependence of solutions of nonlinear structured population models on the model parameters and initial data (Gwiazda et al. 2010; Gwiazda and Marciniak-Czochra 2010; Carrillo et al. 2012); see Appendix for the definition and properties of the flat metric.

Similar results have been recently shown for scalar equations including diffusion; see for instance Barles and Perthame (2008), Barles et al. (2009), Lorz et al. (2011, 2013), Desvillettes et al. (2008), and Lorz et al. (2013) for a model with an additional space structure. The equations studied in Lorz et al. (2013) and Lorz et al. (2013) have been also applied to address cancer heterogeneity, and the influence of the selection process on the cancer resistance to chemotherapy.

The novelty of our work lies in considering a system of two coupled equations. Difficulty of the analysis is related to the specific nonlinearities in the model, which do not allow for component-wise estimates. The proof of boundedness of mass in the scalar equations is based on existence of sub- and supersolutions. In the case of a system, we face a difficulty which appears already in the proof of boundedness of solutions of a structure-independent model. The estimates cannot be concluded directly from the equations. To tackle this problem, we investigate the dynamics of the quotients of solutions of the two variables. Systems of equations also cause additional difficulties when analysing the long-term dynamics in comparison to the scalar equations due to the lack of a rich class of entropies. Convergence to a stationary positive Radon measure has been previously studied for a scalar integro-differential equation which is linear in the nonlocal term as in Jabin and Raoul (2011). This is often referred to as the Evolutionarily Stable Distribution. To deal with model nonlinearities, we make use of a Lyapunov function established previously for a finite dimensional counterpart of the model in Getto et al. (2013) and we show that the total masses of solutions tend asymptotically to the same equilibria.

A system of two equations describing selection and mutation in a stage-structured population has been investigated in Calsina and Cuadrado (2004) and Calsina and Cuadrado (2005) in the context of adaptive dynamics. Analysis of that model is based on a specific structure of nonlinearities appearing only in the mortality terms. Using irreducibility of the mutation operator and the infinite dimensional version of the Perron–Frobenius Theorem, it has been shown that solutions of the model converge to a stationary distribution, which concentrates at the point of maximum fitness in the case of the frequency of mutations tending to zero. The nonlinearity in our model is related to the growth term, which requires a different approach to the analysis of the asymptotic behaviour of the model solutions. The difference in the structure of nonlinear feedbacks is related to a different biological definition of the described processes. While the classical juvenile-adult dynamics is based on a loop of two positive feedbacks and no self-enhancement, the model of cell differentiation involves a negative feedback and a self-enhancement of the first population. Interestingly, the two-stage structure in our model yields stabilisation of the total populations, while even in the basic juvenile-adult models, the two-stage structure may lead to multiple attractors and limit cycles; see for example Baer et al. (2006).

The paper is organised as follows: in Sect. 2, the main results are stated. Analytical results are illustrated by numerical simulations. Proofs of boundedness and strict positivity of the total masses and of the exponential decay of the model solutions outside the set corresponding to the maximal value of the self-renewal parameter are presented in Sect. 3. Section 4 contains the proof of mass convergence to a globally stable equilibrium. Finally, the asymptotic dynamics of the model solutions is shown in Sect. 5. Additionally, in Sect. 6, we show how to extend the analysis of our model to the framework of positive Radon measures with a suitable metric. Finally, in Section 7 we discuss biological conclusions and ideas stemming from this work. A summary of properties of the metrics used in Sect. 5 is provided in the Appendix.

## Main results

We consider the following system of integro-differential equations

3 $$\begin{aligned} \frac{\partial }{\partial t} u_1(t,x)= & {} \left( \frac{2a(x)}{1+K\rho _2(t)}-1\right) pu_1(t,x), \nonumber \\ \frac{\partial }{\partial t} u_2(t,x)= & {} 2\left( 1-\frac{a(x)}{1+K\rho _2(t)}\right) pu_1(t,x) - d u_2(t,x), \\ u_1(0,x)= & {} u_1^0(x),\nonumber \\ u_2(0,x)= & {} u_2^0(x),\nonumber \end{aligned}$$

where

$$\begin{aligned} \rho _i(t) = \int \limits _{\varOmega } u_i (t,x) \,\mathrm {d}x,\quad \text {for} \quad i=1,2 \end{aligned}$$

and $\varOmega \subset \mathbb {R}$ is open and bounded.

In the remainder of this work we make the following assumptions on the model parameters and initial data.

### **Assumption 1**

(i) $a\in C(\overline{\varOmega })$ with $0< a < 1$ and $\overline{\varOmega }$ being a closure of $\varOmega$.
(ii) *p*, *d* and *K* are positive constants.
(iii) $u_1^0, u_2^0 \in L^1(\varOmega )$ are strictly positive a.e. with respect to the Lebesgue measure, i.e. $\int _{B }u_i^0 \mathrm {d}x>0$, for every set B such that $\mathcal{L}^1(B)>0$, $i=1,2$.
(iv) The set of maximal values of the self-renewal parameter *a*, i.e.
  4 $$\begin{aligned} \varOmega _a=\arg \max \limits _{x\in \overline{\varOmega }}a(x) = \left\{ \bar{x}\in \overline{\varOmega }\left| {\bar{a}}:= a(\bar{x}) = \max \limits _{x\in \overline{\varOmega }} a(x)\right. \right\} \end{aligned}$$
  either consists of a single point or it is a set with a positive Lebesgue measure.

### *Remark 1*

The assumption (iv) on the self-renewal fraction *a*(*x*) is made to streamline the presented analysis. If $\varOmega _a$ consists of several isolated points, then the solution is attracted by a finite dimensional subspace spanned by Dirac deltas located at the maximum points of *a*; see Fig. 3. However, in this case the exact pattern may also depend on the shape of function *a*(*x*) near its maximal points. Since analysis of this case requires stronger assumptions on regularity of the initial data and the function *a*(*x*), we consider it separately in Theorem 3.

**Fig. 2:**
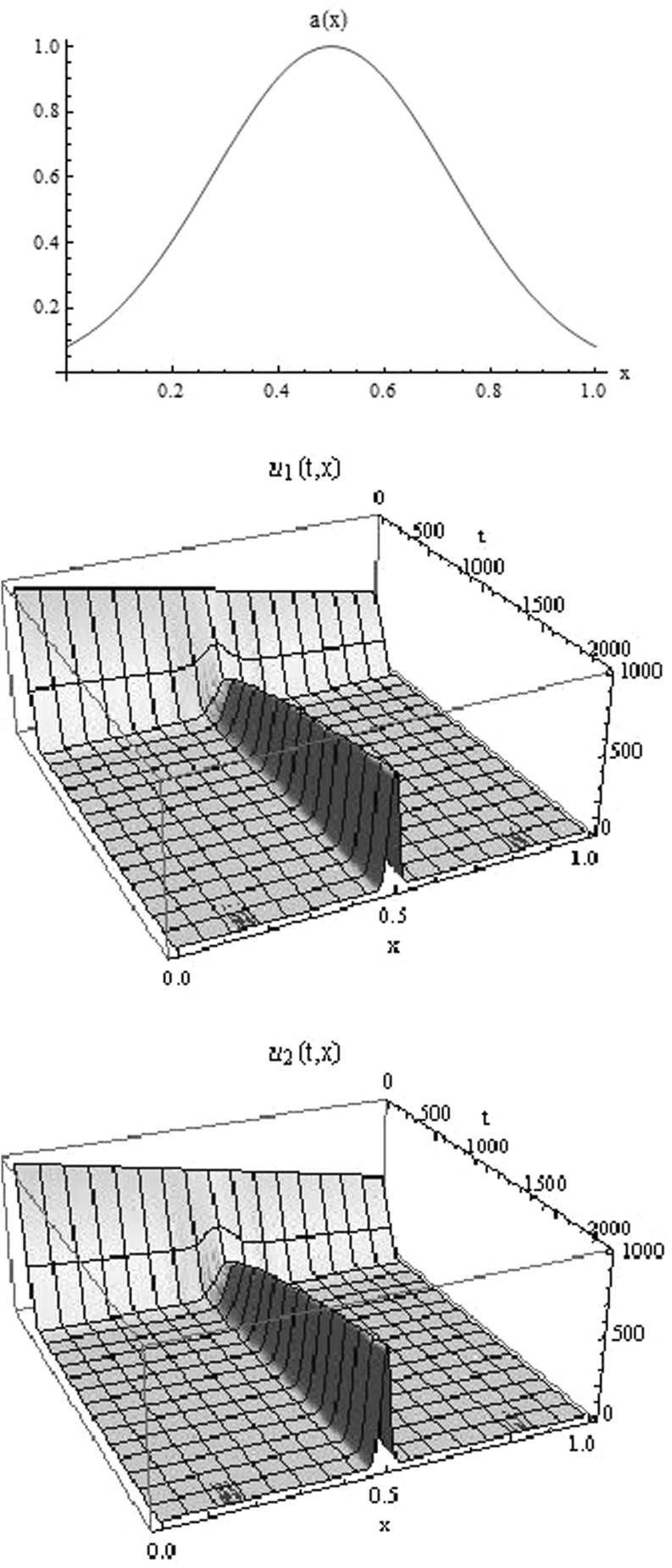
Numerical simulations of the model (3) with the self-renewal function *a*(*x*) having a single local maximum (shown in the *upper panel*). Parameters used in the simulation: $K=0.01$, $p=1$, $d=0.2$ and the initial data: $u_1^0(x) = 1000-500x$, $u_2^0(x) = 1000x^2$. We observe mass concentration in the point ${\bar{x}} =\arg \max \nolimits _{x\in \varOmega }a(x)$ and convergence of the mass to a stable stationary value

**Fig. 3:**
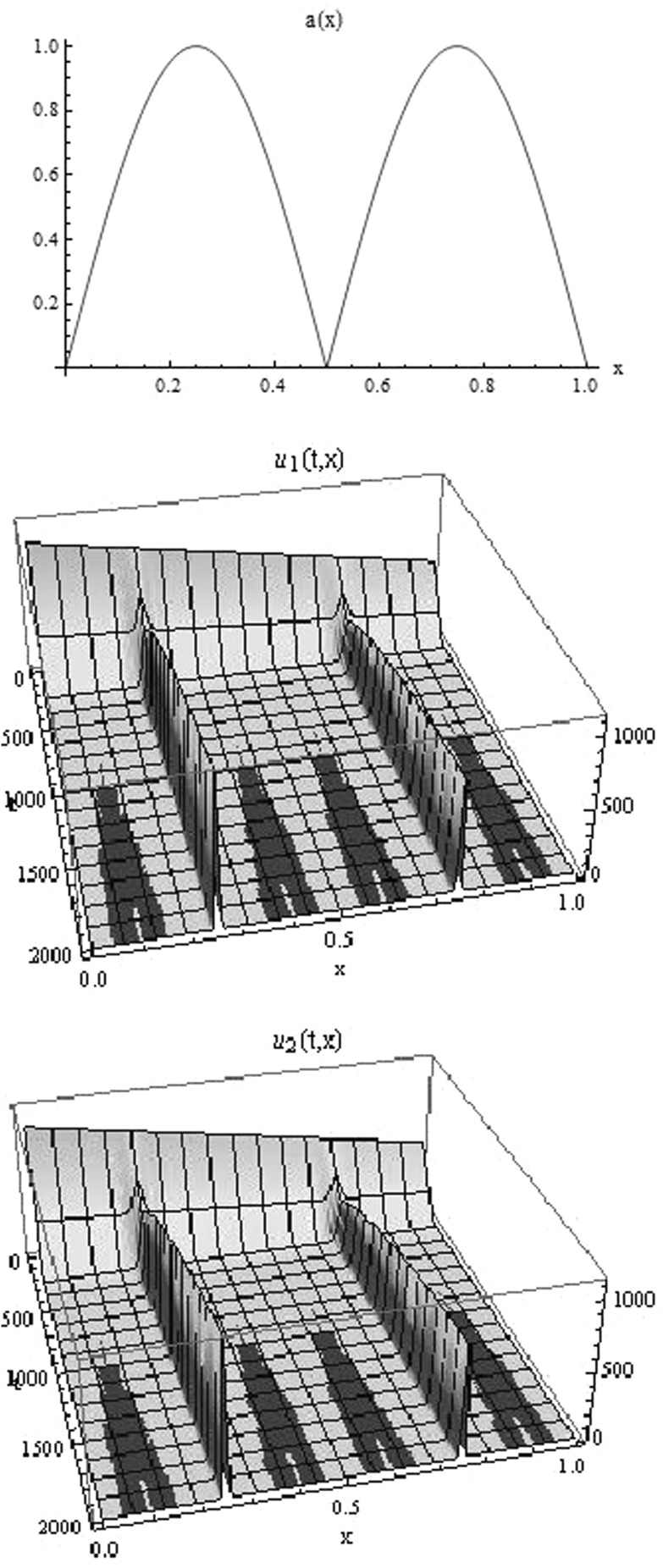
Numerical simulations of the model (3) with the self-renewal function *a*(*x*) having two equal local maxima (shown in the *upper panel*) and the parameters the same as in Fig. 2. We observe mass concentration in two points corresponding to the maximum of the function *a*(*x*) with unequal distribution of the mass between the two points

Existence and uniqueness of a classical solution $u = (u_1, u_2)\!\in \! C^1([0,T), L^1(\varOmega )\times L^1(\varOmega ))$ follow by the standard theory of ordinary differential equations in Banach spaces. More delicate is the question of asymptotic behaviour of the solutions of system (3). Our goal is to show that the solution *u* tends asymptotically to a stationary measure, as it is observed in the numerical simulations, see Figs. 2 and 3. The phenomenon is characterised by the following Theorem.

### **Theorem 1**

Let Assumptions 1 hold and let $(u_1,u_2)$ be a solution of system (3) with initial data $(u_1^0,u_2^0)$. Then, $u_1$ and $u_2$ converge to stationary measures with supports contained in the set $\varOmega _a$ defined in expression (4), as *t* tends to infinity. Moreover,

(i) If $\varOmega _a$ consists of a single point ${\bar{x}}$ and ${\bar{a}}= \max \nolimits _{x\in \overline{\varOmega }} a(x) > \frac{1}{2}$, then the solution converges to a stationary measure (Dirac measure multiplied by a positive constant $(c_1,c_2)=\left( \frac{d}{p}\frac{2{\bar{a}}-1}{K},\frac{2{\bar{a}}-1}{K}\right)$) concentrated in ${\bar{x}}$. Convergence holds in the flat metric (bounded Lipschitz distance); see Appendix for the definition and properties of the bounded Lipschitz distance.
(ii) If $\varOmega _a$ is a set with positive measure and ${\bar{a}}= \max \nolimits _{x\in \overline{\varOmega }} a(x) > \frac{1}{2}$, then the solution converges to a stationary $L^1$-function, such that $\lim \nolimits _{t\rightarrow +\infty }u_i(t,x)= \tilde{c}_i u_i^0(x) {\mathbf {1}}_{\varOmega _a}$, for $i=1,2$, where ${\mathbf {1}}_{\varOmega _a}$ is the characteristic function of the set $\varOmega _a$, $\tilde{c}_1=\frac{d}{p}\frac{(2 {\bar{a}}-1)}{Ku_1^0 |\varOmega _a|}$, and $\tilde{c}_2=\frac{(2 {\bar{a}}-1)}{Ku_1^0 |\varOmega _a|}$. Convergence is strong in $L^1(\varOmega )$.
(iii) If ${\bar{a}} = \max \nolimits _{x\in \overline{\varOmega }} a(x) \le \frac{1}{2}$, then the solution converges to zero, i.e. $\lim \limits _{t\rightarrow +\infty }u_i(t,x)=0$, for $i=1,2$. Convergence is strong in $L^1(\varOmega )$.

### *Remark 2*

If $a(x)\le \frac{1}{2}$ for some points $x \in \varOmega$, then the solutions of the model converge point-wise to zero, i.e. $\lim _{t\rightarrow \infty }(u_1(t,x),u_2(t,x)) =(0,0)$ for every $x \in \varOmega _-:=\{x\in \varOmega \left| a(x)\le \frac{1}{2} \right\}$. This is a straightforward consequence of Eq. (3), since $\rho _2$ is strictly positive, as shown in Lemma 1, and hence $\left( \frac{2a(x)}{1+K\rho _2(t)}-1\right) <0$ for $x \in \varOmega _-$. Therefore, we are interested in evolution of the system for $x \in \varOmega _+:=\varOmega {\setminus } \varOmega _-$. Subpopulations with $a(x) \le \frac{1}{2}$ may affect short-term dynamics of the system; however they have no influence on the asymptotic behaviour.

Details of the proof are presented in Sects. 3, 4 and 5. The proof is based on the following key steps:

*Step 1.* Uniform boundedness and strict positivity of masses $\rho _i(t) = \int \nolimits _{\varOmega } u_i (t,x) \,\mathrm {d}x$ for $i=1,2$ (Lemma 1).

### **Lemma 1**

Let Assumptions 1 (i)–(iii) hold with ${\bar{a}} = \max \nolimits _{x\in \overline{\varOmega }} a(x) > \frac{1}{2}$ and let $(u_1, u_2)$ be a solution of system (3). Then, $\rho _1$ and $\rho _2$ are uniformly bounded and strictly positive, i.e. there exists a positive lower bound, uniform in time.

Proof of this lemma is deferred to Sect. 3.1.

*Step 2.* Exponential extinction of solutions in points outside the set $\varOmega _a$ (Lemma 3).

We start with characterising the asymptotic behaviour of the ratios of solutions taken at different *x* points.

### **Lemma 2**

Let $x_1, x_2\in \overline{\varOmega }$ such that $a(x_1) -a(x_2) < 0$. Then, there exists a constant $M_3>0$ such that

$$\begin{aligned} \frac{u_1(t,x_1)}{u_1(t,x_2)} \le \frac{u_{1}^0(x_1)}{u_{1}^0(x_2)}e^{p\frac{2\left( a(x_1) - a(x_2)\right) }{1+KM_3}t} \,\, \mathop {\longrightarrow }\limits ^{t\rightarrow \infty } \,\,0, \end{aligned}$$

a.e. with respect to the Lebesgue measure.

The proof of this lemma is deferred to Sect. 3.2.

Lemma 2 yields the following result:

### **Corollary 1**

Let $x_1, x_2\in \overline{\varOmega }$ such that $a(x_1)=a(x_2)$. Then, $\frac{u_1(t,x_1)}{u_1(t,x_2)}$ is constant in time.

As a consequence of Lemma 2 we also obtain

### **Lemma 3**

Suppose that Assumptions 1 (i)– (iii) hold. Then, $u(t,x)\rightarrow 0$, exponentially, as $t\rightarrow \infty$ for $x \notin \varOmega _a$ a.e. with respect to the Lebesque measure.

The corresponding proof is presented in Sect. 3.2.

*Step 3.* Convergence of solutions to stationary measures.

Convergence to the stationary solutions follows from the property of the total masses of the solutions $(\int _{\varOmega }u_1(t,x) \mathrm {d}x,\int _{\varOmega }u_2(t,x) \mathrm {d}x)$. We show that if ${\bar{a}} = \max \nolimits _{x\in \overline{\varOmega }} a(x) > \frac{1}{2}$, then the solutions converge to the stationary state of the system with ${\bar{a}}=\max \nolimits _{x\in \overline{\varOmega }}a(x)$.

### **Theorem 2**

Suppose that Assumptions 1 hold, ${\bar{a}} = \max \limits _{x\in \overline{\varOmega }} a(x) > \frac{1}{2}$ and $(\rho _1,\rho _2)= (\int _{\varOmega }u_1(\cdot ,x)\mathrm {d}x,\int _{\varOmega }u_2(\cdot ,x) \mathrm {d}x)$ be total masses of solutions of (3). It holds that $(\rho _1(t),\rho _2(t))\rightarrow (\bar{\rho }_1,\bar{\rho }_2),$ as $t\rightarrow \infty$, where $(\bar{\rho }_1,\bar{\rho }_2)$ are stationary solutions of the corresponding ordinary differential equations model with the maximal value of the self-renewal parameter ${\bar{a}}$, i.e.,

5 $$\begin{aligned} 0= & {} \left( \frac{2{\bar{a}}}{1+K \bar{\rho }_2}-1\right) p \bar{\rho }_1, \nonumber \\ 0= & {} 2\left( 1-\frac{{\bar{a}}}{1+K \bar{\rho }_2}\right) p \bar{\rho }_1 - d \bar{\rho }_2. \end{aligned}$$

Direct calculations based on Eq. (5) yield

### **Corollary 2**

Total masses converge to the values $\bar{\rho }_1=\frac{d}{p}\frac{2{\bar{a}}-1}{K}$ and $\bar{\rho }_2=\frac{2{\bar{a}}-1}{K}$.

Details of the proof of mass convergence are deferred to Sect. 4.

If $\varOmega _a$ consists of a single point ${\bar{x}}$ and ${\bar{a}}= \max \nolimits _{x\in \overline{\varOmega }} a(x) > \frac{1}{2}$, then the exponential decay of the solutions outside the set $\varOmega _a$ together with the convergence of total masses, yields convergence of the solutions to a stationary measure concentrated at ${\bar{x}}$ (a Dirac measure multiplied by a positive constant). In the case of $\varOmega _a$ having a positive Lebesgue measure, convergence of solutions together with Corollary 1 on the stationary distribution of masses among different domain points yields convergence of solutions to the stationary equilibrium. Further details of the proof of convergence of solutions to the stationary measures are given in Sect. 5.

### *Remark 3*

In the case $\varOmega _a=\{ {\bar{x}}\}$, the convergence holds in the weak$*$ topology of Radon measures. In general, we cannot expect the strong (norm- total variation) convergence of the solution to a stationary solution. If the set $\varOmega _a \subset \mathbb {R}$ has zero Lebesgue measure and consists of a single point [compare Assumptions 1 (iv)], then the model solutions for any finite time point are uniformly continuous with respect to the Lebesgue measure and $u_i(t,\cdot ) \mathcal L^1 \rightarrow c_i \delta _{{\bar{x}}}$, weakly$*$, for $i=1,2$. Here, $u_i(t,\cdot )\mathcal L^1$ denotes the measure such that *u* is its Radon–Nikodym derivative with respect to $\mathcal L^1$.

Hence, the distance between the two solutions $TV(u_i(t,\cdot ),c_i\delta _{\bar{x}})\ge 2c_i$. The problem can be solved by considering convergence with respect to a suitable metric, for example the flat metric (bounded Lipschitz distance); for details see Sect. 5.

If the support of ${\bar{a}}$ is not a single point set, then the stationary distribution of masses depends on the initial conditions. If $\varOmega _a$ has a positive Lebesgue measure, then the distribution of masses results from Corollary 1. If $\varOmega _a$ consists of a discrete set of points, then the stationary solution takes the form of a linear combination of Dirac deltas; see Fig. 3. We show that in such case the limit function depends on the shape of *a*(*x*) in the neighbourhood of the concentration points.

### **Theorem 3**

(Co-existence of different stationary solutions) Let Assumptions 1 (i)–(iii) hold and, additionally, the initial functions $u_1^0, u_2^0 \in C(\varOmega )$. Let the set $\varOmega _a$ of the maximum values of the self-renewal parameter *a* (as defined in expression (4)) consist of two points $\varOmega _a=\{{\bar{x}}_1, {\bar{x}}_2\}$ and $u_1^0$ be strictly positive on $\varOmega _a$. Then,

(i) If there exists a diffeomorphism $\varPhi \in C^1(U_1)$, where $U_1$ is an open neighbourhood of ${\bar{x}}_1$, such that
  6 $$\begin{aligned} \varPhi ({\bar{x}}_1)= & {} {\bar{x}}_2,\nonumber \\ a(x)= & {} a(\varPhi (x))\quad \text {for all} \quad x\in U_1, \end{aligned}$$
  then solutions $(u_1,u_2)$ of system (3) converge to stationary measures, which are linear combinations of Dirac measures concentrated in ${\bar{x}}_1$ and ${\bar{x}}_2$, multiplied by strictly positive constants.
(ii) If the mapping $\varPhi$ with the properties defined by condition (6) is only a homeomorphism with a singular Jacobian of the inverse mapping $\varPhi ^{-1}$ at ${\bar{x}}_2$, then solutions $(u_1,u_2)$ of system (3) converge to stationary measures concentrated in ${\bar{x}}_2$.

The proof of this theorem is deferred to Sect. 5.

### *Remark 4*

If *a* is an analytic function and $\varOmega \subset \mathbb {R}$, then a diffeomorphism satisfying condition (6) exists if the first nonconstant nonzero terms of Taylor expansion of the function *a*(*x*) are of the same order.

This observation suggests how to construct *a*(*x*) with $\varOmega _a=\{{\bar{x}}_1, {\bar{x}}_2\}$ such that solutions extinct at one of the points of $\varOmega _a$. For example, we may define *a*(*x*) with $x\in \varOmega =[0,1]$ such that

$$\begin{aligned} a(x):=\left\{ \begin{array}{ccc} - (x-\frac{1}{4})^2 + \frac{9}{10} &{}\quad for\, x\in [0, \frac{3}{8}),\\ - (x-\frac{3}{4})^4+\frac{9}{10} &{}\quad for\, x\in (\frac{5}{8}, 1]. \end{array}\right. \end{aligned}$$

and a smooth extension of *a*(*x*) on the interval $(\frac{3}{8},\frac{5}{8})$ satisfying $0<a(x)<1$. We obtain $\varOmega _a=\{\frac{1}{4},\frac{3}{4}\}$, and a mapping $\varPhi (x)=\sqrt{x-\frac{1}{4}}+\frac{3}{4}$ satisfying condition (6) on $U_1=(\frac{1}{4}-\varepsilon ,\frac{1}{4}+\varepsilon )$, where $\varepsilon <\frac{1}{8}$. Consequently, $\varPhi ^{-1}(x)= (x-\frac{3}{4})^2+\frac{1}{4}$ and it is singular at $x=\frac{3}{4}$. Hence, the total mass concentrates at the point $x=\frac{3}{4}$ and there is an extinction of mass at $x=\frac{1}{4}$.

## Proof of mass concentration

### Boundedness and strict positivity of masses

All considerations in this Section hold for $x\in \varOmega$ a.e. with respect to the Lebesque measure.

First, we notice that the solutions $(u_1,u_2)$ are nonnegative, since $a(x)/(1+K\rho _2)<1$. Before proving Lemma 1, we show the following technical result.

#### **Lemma 4**

Under the assumptions of Lemma 1, the function $U = \frac{u_1}{u_2}$ is uniformly bounded on $\varOmega \times \mathbb {R}^+$.

#### *Proof*

The equation for $U(t,x) = \frac{u_1(t,x)}{u_2(t,x)}$ reads for $t>0$

7 $$\begin{aligned} \frac{\partial }{\partial t} U(t,x)= & {} U(t,x)\Biggr (p\left( \frac{2a(x)}{1+K\rho _2(t)}-1\right) +d \nonumber \\&-2p\left( 1 - \frac{a(x)}{1+K\rho _2(t)}\right) U(t,x)\Biggr ). \end{aligned}$$

Since

$$\begin{aligned} p\left( \frac{2a(x)}{1+K\rho _2(t)}-1\right) +d \le 2p{\bar{a}} +d \end{aligned}$$

and

$$\begin{aligned} 1 - \frac{a(x)}{1+K\rho _2(t)}>1-{\bar{a}}, \end{aligned}$$

and the right-hand side of Eq. (7) is a logistic type nonlinearity, we conclude that

$$\begin{aligned} U(t,x) \le \max \left\{ U(0,x),\frac{2p\bar{a} +d}{2p(1-\bar{a})}\right\} =: M_1 \quad \forall \; (t,x) \in [0,T)\times \varOmega . \end{aligned}$$

By definition of *U*, we can infer that

$$\begin{aligned} u_1(t,x) \le M_1 u_2(t,x) \quad \forall \; (t,x)\in [0,T)\times \varOmega . \end{aligned}$$

As a straightforward consequence of Lemma 4, we deduce

#### **Corollary 3**

Under the assumptions of Lemma 1, it holds

8 $$\begin{aligned} \int \limits _{\varOmega } u_1(t,x)\,\mathrm {d}x \le M_1 \int \limits _{\varOmega } u_2(t,x)\,\mathrm {d}x = M_1 \rho _2(t). \end{aligned}$$

Now we state another technical result in the spirit of Lemma 4.

#### **Lemma 5**

There exist constants $M_4>0$ and $0<\gamma <1$ such that $\rho _2(t) \le M_4\rho _1^{\gamma }(t)$ for all $t\ge 0$.

#### *Proof*

Calculating the derivative of the quotient of $\rho _2(t)$ and $\rho _1^\gamma (t)$, we obtain

$$\begin{aligned} \frac{d}{d t} \frac{\rho _2(t)}{\rho _1^{\gamma }(t)}= & {} \frac{\frac{d}{d t} \rho _2(t) \rho _1^{\gamma }(t) - \rho _2(t) \gamma \rho _1^{\gamma -1}(t)\frac{d}{dt }\rho _1(t)}{\rho _1^ {2\gamma }(t)} \\= & {} \frac{\int _{\varOmega }\left( 2(1-\frac{a(x)}{1+K\rho _2(t)})pu_1(t,x) -d u_2(t,x)\right) \,\mathrm {d}x}{\rho _1^{\gamma }(t)} \\&-\, \frac{\rho _2(t)}{\rho _1^{\gamma }(t)}\frac{\gamma \int _{\varOmega } \left( \frac{2a(x)}{1+K\rho _2(t)} - 1\right) pu_1(t,x)\, \mathrm {d}x}{\rho _1} \\\le & {} \frac{\int _{\varOmega }\left( 2 (1- \frac{a(x)}{1+K\rho _2(t)})pu_1(t,x) -d u_2(t,x) \right) \,\mathrm {d}x}{\rho _1^{\gamma }(t)} + \frac{\rho _2(t)}{\rho _1^ {\gamma }(t)}\gamma p \\\le & {} 2p\rho _1^{1-\gamma }(t) + \frac{\rho _2(t)}{\rho _1^{\gamma }(t)}(\gamma p-d) \le 2pM_2^{1-\gamma } + \frac{\rho _2(t)}{\rho _1^{\gamma }(t)}(\gamma p -d). \end{aligned}$$

This estimate holds for arbitrary $\gamma \in (0,1)$, so in particular for those satisfying $\gamma p -d < 0$. Arguing as before, we deduce that, for all $t\ge 0$,

9 $$\begin{aligned} \frac{\rho _2}{\rho _1^{\gamma }}(t) \le \max \left\{ \frac{\rho _2(0)}{\rho _1^{\gamma }(0)}, \frac{2pM_2^{1-\gamma }}{d - \gamma p}\right\} =: M_4. \end{aligned}$$

Equipped with Lemmas 4 and 5, we prove Lemma 1.

#### *Proof (of Lemma 1)*

(i) First, we show uniform boundedness of masses $\rho _1$ and $\rho _2$, which yields also the global existence of solutions $(u_1, u_2)\in C^1([0,\infty ), L^1(\varOmega )\times L^1(\varOmega ))$.

To show boundedness of $\rho _1$, we apply inequality (8) to the first equation of system (3)

$$\begin{aligned} \frac{\partial }{\partial t} u_1(t,x)= & {} \left( \frac{2a(x)}{1+K\rho _2(t)}-1\right) pu_1(t,x) \le \left( \frac{2a(x)}{1+ \frac{K}{M_1}\rho _1(t)}-1\right) pu_1(t,x) \\\le & {} \left( \frac{2\bar{a}}{1+\frac{K}{M_1}\rho _1(t)}-1\right) pu_1(t,x). \end{aligned}$$

Integrating this inequality over $\varOmega$ yields

10 $$\begin{aligned} \frac{d}{d t} \rho _1(t) \le \left( \frac{2\bar{a}}{1+\frac{K}{M_1}\rho _1(t)}-1\right) p\rho _1(t). \end{aligned}$$

Using a similar argument as in the proof of Lemma 4, we conclude that

11 $$\begin{aligned} \rho _1(t) \le \max \left\{ \rho _1(0),\frac{(2\bar{a}-1)M_1}{K}\right\} =: M_2. \end{aligned}$$

Boundedness of $\rho _2$ results from the second equation of system (3), nonnegativity of $\rho _2$ and the assumptions on *a*. It holds

$$\begin{aligned} \frac{\partial }{\partial t} u_2(t,x)= & {} 2\left( 1-\frac{a(x)}{1+K\rho _2(t)}\right) pu_1(t,x) -d u_2(t,x) \le 2pu_1(t,x) -d u_2(t,x). \end{aligned}$$

Integrating over $\varOmega$ and using (11), we obtain

$$\begin{aligned} \frac{d}{d t} \rho _2(t) \le 2p\rho _1(t) -d\rho _2(t) \le 2pM_2 -d\rho _2(t). \end{aligned}$$

Hence, we conclude that

12 $$\begin{aligned} \rho _2(t) \le \max \left\{ \rho _2(0),\frac{2pM_2}{d}\right\} =: M_3. \end{aligned}$$

(ii) We show that masses $\rho _1$ and $\rho _2$ have a strictly positive lower bound, uniform in time.

We estimate the growth of $\rho _1$ using a decomposition of the domain $\varOmega =\varOmega _- + \varOmega _+$, where $\varOmega _-:=\{x\in \varOmega \left| a(x)\le \frac{1}{2} \right\}$ and $\varOmega _+:=\{x\in \varOmega \left| a(x)> \frac{1}{2} \right\}$.

First, we assume that the set $\varOmega _-$ is nonempty, i.e. $\int _{\varOmega _-} u_1^0(x)>0$. We denote

$$\begin{aligned} \rho _1^-(t)=\int \limits _{\varOmega _-} u_1(t,x)\; \mathrm {d}x \quad \text {and} \quad \rho _1^+(t)=\int \limits _{\varOmega _+} u_1(t,x)\; \mathrm {d}x. \end{aligned}$$

Using the explicit form of the solution

13 $$\begin{aligned} u_1(t,x)=u^0_1(x)e^{\int \limits _0^t\left( \frac{2a(x)}{1+K\rho _2(\tau )}-1\right) p \;\mathrm {d}\tau } \end{aligned}$$

and the properties of the function *a*(*x*) on the two subdomains, we obtain

14 $$\begin{aligned} \frac{\rho _1^+(t)}{\rho _1^-(t)}= & {} \frac{\int _{\varOmega _+} u_1^0(x) e^{\int _0^t \left( \frac{2a(x)}{1+K\rho _2(\tau )} -1\right) p\;\mathrm {d}\tau }\,\mathrm {d}x}{\int _{\varOmega _-} u_1^0(x) e^{\int _0^t \left( \frac{2a(x)}{1+K\rho _2(\tau )} -1\right) p\; \mathrm {d}\tau }\,\mathrm {d}x} \ge \frac{ \inf _{\varOmega _+} e^{\int _0^t \left( \frac{2a(x)}{1+K\rho _2(\tau )} -1\right) p\;\mathrm {d}\tau }\,\int _{\varOmega _+} u_1^0(x) \;\mathrm {d}x}{ \sup _{\varOmega _-} e^{\int _0^t \left( \frac{2a(x)}{1+K\rho _2(\tau )} -1\right) p\; \mathrm {d}\tau }\, \int _{\varOmega _-} u_1^0(x)\;\mathrm {d}x}\nonumber \\= & {} \frac{ e^{\int _0^t \left( \frac{1}{1+K\rho _2(\tau )} -1\right) p\;\mathrm {d}\tau }\,\int _{\varOmega _+} u_1^0(x) \;\mathrm {d}x}{ e^{\int _0^t \left( \frac{1}{1+K\rho _2(\tau )} -1\right) p\; \mathrm {d}\tau }\, \int _{\varOmega _-} u_1^0(x)\;\mathrm {d}x}=\frac{\rho _1^+(0)}{\rho _1^-(0)}. \end{aligned}$$

Combining estimates (9) and (14) yields

15 $$\begin{aligned} \rho _2(t) \le M_4(\rho _1^+(t) + \rho _1^-(t))^{\gamma }\le M_4 \left( \rho _1^+(t)\left( 1+ \frac{\rho _1^+(0)}{\rho _1^-(0)} \right) \right) ^{\gamma }=M_5 \left( \rho _1^+(t)\right) ^{\gamma } \end{aligned}$$

with $$M_5=M_4 \left( 1+ \frac{\rho _1^+(0)}{\rho _1^-(0)} \right) ^{\gamma }$$.

With estimate (15) at hand, we show that $\rho _1^+$ is strictly positive for every $t\in \mathbb {R}^+$. We estimate its dynamics

$$\begin{aligned} \frac{d}{d t} \rho _1^+(t)= & {} \int \limits _{\varOmega _+} \left( \frac{2a(x)}{1+K\rho _2(t)} -1\right) pu_1(t,x)\,\mathrm {d}x\\\ge & {} \left( \frac{2\underline{a}}{1+KM_5\left( \rho _1(t)^+\right) ^{\gamma }} -1\right) p\rho _1(t), \end{aligned}$$

where $$\underline{a}= \min \limits _{x\in \overline{\varOmega }_+}a(x)>\frac{1}{2}$$.

The term in the brackets is strictly positive for $\rho _1^+$ small enough, i.e. for

$$\begin{aligned} \rho _1^+(t) \le \left( \frac{2\underline{a} -1}{KM_5}\right) ^{\frac{1}{\gamma }}, \end{aligned}$$

which is a positive constant, since $\underline{a}>\frac{1}{2}$.

Hence, we obtain the estimate

$$\begin{aligned} \rho _1(t) \ge \min \Big \{\rho _1(0), \left( \frac{2\underline{a} -1}{KM_5}\right) ^{\frac{1}{\gamma }}\Big \} =: M_6 \quad \forall \; t \in [0,\infty ). \end{aligned}$$

Consequently, we obtain the strict positivity of $\rho _1$ and using the second equation of (3), also the strict positivity of $\rho _2$. In the case of $\varOmega _-=\emptyset$, it holds $\rho _1=\rho _1^+$ and the proof is complete if we set $M_5=M_4$.

### Asymptotic behaviour of the solutions

In the next step, we show that the first component of the solution of system (3) tends to zero for ${\bar{x}} \notin \varOmega _a$ a.e. with respect to the Lebesque measure.

#### *Proof (of Lemma 2)*

We choose two points $x_1, x_2\in \overline{\varOmega }$ such that $a(x_1) -a(x_2) < 0$, and calculate

$$\begin{aligned} \frac{\partial }{\partial t} \frac{u_1(t,x_1)}{u_1(t,x_2)} = p\frac{u_1(t,x_1)}{u_1(t,x_2)}\left( 2\frac{a(x_1)-a(x_2)}{1+K\rho _2(t)}\right) \le p\frac{u_1(t,x_1)}{u_1(t,x_2)}\left( 2\frac{a(x_1)-a(x_2)}{1+KM_3}\right) . \end{aligned}$$

Solving the above differential inequality for $\frac{u_1(t,x_1)}{u_1(t,x_2)}$, we obtain the assertion of this Lemma by the choice of $x_1$ and $x_2$.

#### **Lemma 6**

Let $x_1,x_2 \in \overline{\varOmega }$ be such that $a(x_1) - a(x_2) < 0$, then

$$\begin{aligned} \frac{u_2(t,x_1)}{u_2(t,x_2)} \mathop {\longrightarrow }\limits ^{t\rightarrow \infty } 0, \end{aligned}$$

a.e. with respect to the Lebesque measure.

#### *Proof*

We use a similar ansatz as in Lemma 2 and calculate for $t>0$

$$\begin{aligned} \frac{\partial }{\partial t} \frac{u_2(t,x_1)}{u_2(t,x_2)}= & {} 2\left( 1-\frac{a(x_1)}{1+K\rho _2(t)}\right) p\frac{u_1(t,x_1)}{u_2(t,x_2)} \\&-2\left( 1-\frac{a(x_2)}{1+K\rho _2(t)}\right) p \frac{u_2(t,x_1)}{u_2(t,x_2)}\frac{u_1(t,x_2)}{u_2(t,x_2)}. \end{aligned}$$

Applying Lemma 2, we obtain

$$\begin{aligned} \frac{\partial }{\partial t} \frac{u_2(t,x_1)}{u_2(t,x_2)}= & {} p\frac{u_1(t,x_2)}{u_2(t,x_2)}\Bigg (2\left( 1-\frac{a(x_1)}{1+K\rho _2}\right) \frac{u_1^0(x_1)}{u_1^0(x_2)}e^{\frac{2\left( a(x_1)-a(x_2)\right) t}{1+KM_3}} \\&- 2\left( 1-\frac{a(x_2)}{1+K\rho _2}\right) \frac{u_2(t,x_1)}{u_2(t,x_2)}\Bigg ). \end{aligned}$$

Thus, we deduce the following bound for $\frac{u_2(t,x_1)}{u_2(t,x_2)}$

$$\begin{aligned} \frac{u_2(t,x_1)}{u_2(t,x_2)} \le \frac{\left( 1-\frac{a(x_1)}{1+KM_3}\right) \frac{u_1^0(x_1)}{u_1^0(x_2)}e^{\frac{2(a(x_1) - a(x_2))t}{1+KM_3}}}{1- a(x_2)}, \end{aligned}$$

where the right hand side tends exponentially to zero, as *t* tends to infinity.

This concludes the proof.

Having shown the dynamics of the ratios of the values of a solution at different *x* points, we prove that the solutions converge to zero outside the set of points with a maximum value of the parameter *a*(*x*).

#### *Proof (of Lemma 3)*

Let $\tilde{x}$ be a point different from ${\bar{x}}$ and assume that $\lim _{t \rightarrow \infty } u(t,\tilde{x}) > 0$. Continuity of *a*(*x*) implies that the set of *x*, such that $a(x)>a(\tilde{x})$, is an open nonempty set and, therefore, it has positive measure. Since Lemma 2 holds for every $x, \tilde{x} \in \overline{\varOmega }$ such that $a(x) - a(\tilde{x}) > 0$, we conclude that *u*(*t*, *x*) tends exponentially to $+ \infty$ for every *x* such that $a(x)>a(\tilde{x})$. This is, however, in contradiction with the uniform boundedness of the mass $\int _{\varOmega }u(t,x)\mathrm {d}x$.

## Proof of convergence of the total mass

We begin the proof of Theorem 2 by showing the following lemma, which allows comparing two dynamical systems.

### **Lemma 7**

Let $t\rightarrow X_F (t,\cdot )$ be a one-parameter family of $C^1$-diffeomorphisms (semiflows) $X_F (t, (0,\infty )\times (0,\infty ))\subset (0,\infty )\times (0,\infty )$, for every $t\ge 0$, generated by the ordinary differential equation

16 $$\begin{aligned} \frac{du}{dt}=F(u) \end{aligned}$$

such that $V\in C^1((0,\infty )\times (0,\infty ))$, with a single minimum $\bar{u}$, is a strict Lyapunov functional, i.e. $\frac{d}{dt}X_F(t,u)|_{t=0}\cdot \nabla V(u) = 0$ for $u=\bar{u}$ and $\frac{d}{dt}X_F(t,u)|_{t=0}\cdot \nabla V(u) < 0$ otherwise. Then, if $\tilde{u}$ is a solution of

17 $$\begin{aligned} \frac{d \tilde{u}}{dt}=F(\tilde{u}) + f, \end{aligned}$$

where $lim_{t\rightarrow \infty }sup_{\tau \in [t,\infty )}|f(\tau )|=0$ and $\overline{\mathrm{Im} (\tilde{u}(\cdot ))}:=\overline{\cup _{t\in [0,\infty )} \{\tilde{u}(t)\}} \subset (0,\infty )\times (0,\infty )$ is compact, then $\tilde{u}(t) \rightarrow \bar{u}$ for $t \rightarrow \infty$.

### *Proof*

For arbitrary $a>\bar{V}$, we define a truncation

$$\begin{aligned} V_a(u):=\left\{ \begin{array}{ccc} V(u)-a &{}\quad \text { if }&{} V(u)\ge a,\\ 0 &{}\quad \text { if }&{} V(u)<a. \end{array}\right. \end{aligned}$$

Since $V_a \in W^{1,\infty }(U)$, where *U* is the intersection of all convex sets containing $\overline{\mathrm{Im}(\tilde{u}(\cdot ))}$, $U={\mathrm{conv}}(\overline{\mathrm{Im}(\tilde{u}(\cdot ))})\subset (0,\infty )\times (0,\infty )$, and $\frac{d}{d t} \tilde{u} \in L^1(\varOmega )$, then we can define the time derivative of $V_a(\tilde{u}(t))$ using the chain rule. $\nabla _{u} V_a$ is defined in a classical sense only outside the set $V( u)=a$, but it has a Clarke derivative, i.e. a generalised subdifferential for a locally Lipschitz function (Clarke 1983), on the set $V=a$. In the following, $\nabla _{\tilde{u}} V_a(\tilde{u})$ is an extension of the classical definition, involving the maximal element of the Clarke derivative, to the set where the classical derivative is not defined.

Let us define $\beta : \overline{\mathrm{Im} V(u)}\rightarrow (0,\infty )$ such that

$$\begin{aligned} \beta (x)=\inf \limits _{\{u\in U| V_a(u)=x\}}\left\{ \frac{d}{dt}X_F(t,u)|_{t=0}\cdot V(u) \right\} . \end{aligned}$$

Since $\beta$ is a continuous function defined on a compact set, it achieves a strictly positive minimum. Furthermore, for the truncation function $V_a$, there exists a positive constant $\tilde{\beta }_a$ such that $\beta (V_a)\ge \tilde{\beta }_a V_a$. Hence, we obtain

18 $$\begin{aligned} \frac{dV_a(\tilde{u}(t))}{dt}\le - \tilde{\beta }_a V_a(\tilde{u}(t))+ \nabla _{\tilde{u}} V_a(\tilde{u}(t)) \cdot f(t). \end{aligned}$$

Using compactness of the set *U*, we estimate $\nabla _{\tilde{u}} V_a(\tilde{u}(t))$ by its $L^{\infty }$ norm, which yields the following inequality,

$$\begin{aligned} \frac{dV_a(\tilde{u}(t))}{dt}\le - \tilde{\beta }_a V_a(\tilde{u}(t))+ C |f(t)|, \end{aligned}$$

where $$C = \left\| \nabla _{\tilde{u}}V\right\| _{L^{\infty }(U)}$$.

Integrating the above estimate, we obtain

19 $$\begin{aligned} V_a(\tilde{u}(t))\le V_a(u_0)e^{-\tilde{\beta }_a t} + C\int _0^t |f(\tau )| e^{-\tilde{\beta }_a (t-\tau )}\mathrm {d}\tau . \end{aligned}$$

We show that the right-hand side of inequality (19) tends to zero for $t \rightarrow \infty$.

$$\begin{aligned}&\int _0^t |f(\tau )| e^{-\tilde{\beta }_a (t-\tau )}\mathrm {d}\tau \nonumber \\&\quad = \int _0^{\frac{t}{2}} |f(\tau )| e^{-\tilde{\beta }_a (t-\tau )}\mathrm {d}\tau +\int _{\frac{t}{2}}^t |f(\tau )| e^{-\tilde{\beta }_a (t-\tau )}\mathrm {d}\tau \\&\quad \le \sup _{\tau \in \mathbb {R}^+}|f(\tau )| \int _0^{\frac{t}{2}} e^{-\tilde{\beta }_a (t-\tau )}\mathrm {d}\tau + \sup _{\tau \in [\frac{t}{2},\infty ]}|f(\tau )| \;\int _{\frac{t}{2}}^t e^{-\tilde{\beta }_a (t-\tau )}\mathrm {d}\tau \\&\quad \le \sup _{\tau \in \mathbb {R}^+}|f(\tau )| \; \frac{1}{\tilde{\beta }_a}\;e^{-\frac{\tilde{\beta }_a t}{2}} \left( 1- e^{-\frac{\tilde{\beta }_a t}{2}}\right) + \sup _{\tau \in [\frac{t}{2},\infty ]}|f(\tau )| \; \frac{1}{\tilde{\beta }_a}\; \left( 1- e^{-\frac{\tilde{\beta }_a t}{2}}\right) . \end{aligned}$$

Since, by assumption $\lim _{t \rightarrow \infty } \sup _{\tau \in [\frac{t}{2},\infty ]}|f(\tau )|=0$, passing to the limit, we obtain

$$\begin{aligned} \lim _{t \rightarrow \infty } \int _0^t |f(\tau )| e^{-\tilde{\beta }_a (t-\tau )}\mathrm {d}\tau =0. \end{aligned}$$

Convergence holds for every *a*, which yields convergence $V(\tilde{u}(t))\rightarrow \bar{V}$, i.e. to the minimum of the function *V*. In turn, this ensures that $\tilde{u}(t)\rightarrow \bar{u}$.

### *Proof (of Theorem 2)*

To apply Lemma 7 to system (3), we consider a finite dimensional model obtained by setting *a*(*x*) to a constant value ${\bar{a}}$

20 $$\begin{aligned} \frac{d}{d t} v_1= & {} \left( \frac{2{\bar{a}}}{1+K v_2}-1\right) p v_1, \nonumber \\ \frac{d}{d t} v_2= & {} 2\left( 1-\frac{{\bar{a}}}{1+K v_2}\right) p v_1 - d v_2, \nonumber \\ v_1(0)= & {} v_1^0,\nonumber \\ v_2(0)= & {} v_2^0. \end{aligned}$$

Note that the above equation generates a $C^1$-semiflow, which is invariant on $(0,\infty ) \times (0,\infty ).$ We check that the two systems (20) and (25) fulfill the assumptions of Lemma 7.

Lyapunov function for system (20) has been previously constructed in Getto et al. (2013). It assumes the form

21 $$\begin{aligned} V(v_1,v_2):=\frac{1}{pG(\bar{v}_2)}V_{1}(v_1)+\frac{1}{d}V_{2}(v_2), \end{aligned}$$

where

$$\begin{aligned} V_{1}(v_1):= & {} \frac{v_1}{\bar{v}_{1}}-1-\ln \frac{v_1}{\bar{v}_1},\\ V_{2}(v_2):= & {} \frac{v_2}{\bar{v}_2}-1-\frac{1}{\bar{v}_2}\int _{\bar{v}_2}^{v_2}\frac{G(\bar{v}_2)}{G(\xi )}d\xi , \end{aligned}$$

$(\bar{v}_{1},\bar{v}_{2})$ is the stationary solution, and

22 $$\begin{aligned} G(v_2):=2\left( 1-\frac{{\bar{a}}}{1+kv_2}\right) \quad \text { for } v_2\ge 0. \end{aligned}$$

Lyapunov function (21) is well-defined for every $(v_1,v_2)\in (0,\infty )\times (0,\infty )$. Moreover, $V\in C^{\infty }(0,\infty )\times (0,\infty )$.

Note that $V_1(v_1)$ is strictly convex and therefore $\frac{\partial }{\partial v_1} V_1 \not = 0$ for $v_1\not = \bar{v}_1$. Similar observation holds for $V_2(v_2)$. Hence $(\bar{v}_1,\bar{v}_2)$ is the global minimum of the Lyapunov function.

Direct calculations, as provided in Getto et al. (2013), allow to check that

23 $$\begin{aligned} \frac{d}{dt}V(v_1(t),v_2(t))\le 0, \end{aligned}$$

for the solutions of system (20). Moreover, the equality $\frac{d}{dt}V(v_1(t),v_2(t))=0$ holds only for the stationary solution $(\bar{v}_1, \bar{v}_2)$.

To show convergence of the total mass of the solution of system (3) to a global equilibrium, we integrate equations (3) with respect to *x* and obtain

24 $$\begin{aligned} \frac{d}{d t} \rho _1(t)= & {} \int _{\varOmega }\left( \frac{2a(x)}{1+K \rho _2(t)}-1\right) p u_1(t,x)\mathrm {d}x, \nonumber \\ \frac{d}{d t} \rho _2(t)= & {} 2\int _{\varOmega } \left( 1-\frac{a(x)}{1+K \rho _2(t)}\right) p u_1(t,x)\mathrm {d}x- d \int _{\varOmega } u_2(t,x) \mathrm {d}x,\nonumber \\ \rho _1(0)= & {} \int _{\varOmega } u_1^0(x) \mathrm {d}x, \nonumber \\ \rho _2(0)= & {} \int _{\varOmega }u_2^0(x) \mathrm {d}x. \end{aligned}$$

This can be rewritten as

25 $$\begin{aligned} \frac{d}{d t} \rho _1(t)= & {} \left( \frac{2 {\bar{a}}}{1+K \rho _2(t)}-1\right) p \rho _1(t) + \frac{2p}{1+K\rho _2(t)} \int _{\varOmega }\left( a(x)-{\bar{a}}\right) u_1(t,x) \mathrm {d}x,\nonumber \\ \frac{d}{d t} \rho _2(t)= & {} 2 \left( 1-\frac{{\bar{a}}}{1+K \rho _2(t)}\right) p \rho _1(t)\nonumber \\&+ \frac{2p}{1+K \rho _2(t)}\int _{\varOmega } \left( {\bar{a}} - a(x)\right) u_1(t,x)\mathrm {d}x -d\rho _2(t), \\ \rho _1(0)= & {} \int _{\varOmega } u_1^0(x) \mathrm {d}x,\nonumber \\ \rho _2(0)= & {} \int _{\varOmega }u_2^0(x) \mathrm {d}x.\nonumber \end{aligned}$$

**Fig. 4:**
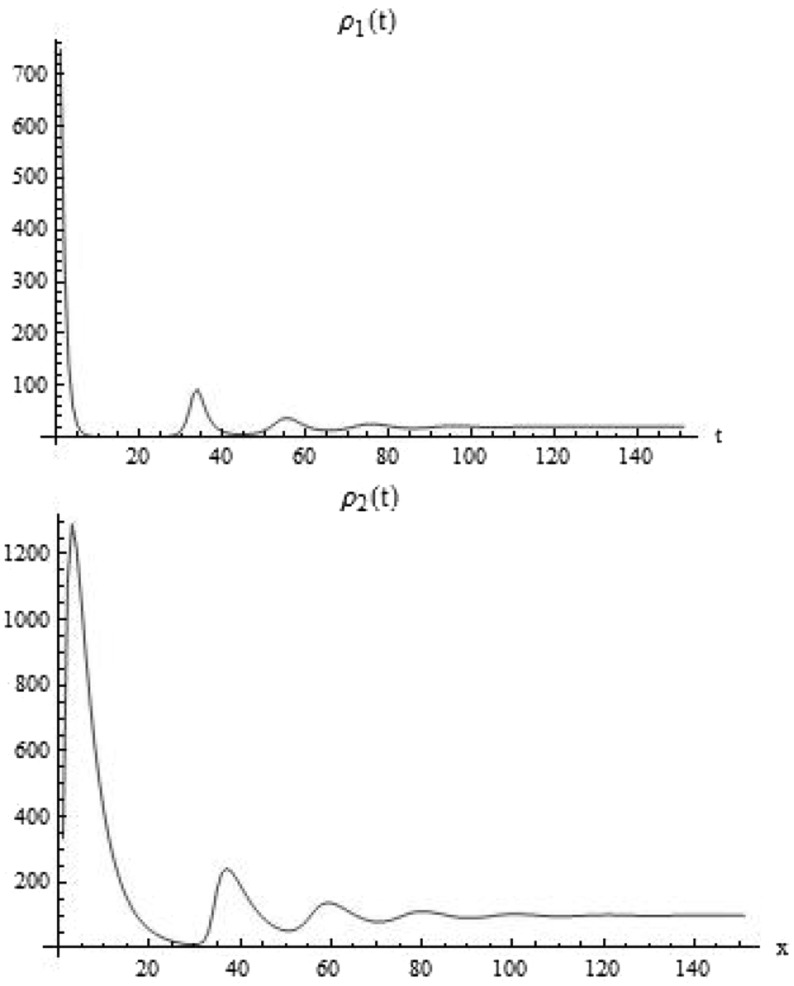
Using the trapezoid rule to approximate the integral of $\rho _1(t),\rho _2(t)$, we observe numerically the convergence of the total mass to a constant value. The parameter set is the same as in Fig. 2

By Lemma 1, $\overline{\mathrm{Im}} ((\rho _1(\cdot ),\rho _1(\cdot ))\subset (0,\infty )\times (0,\infty )$ and it is compact (see Lemma 1 and Fig. 4).

To show that the perturbation function on the right-hand side converges to zero as $t\rightarrow \infty$, we calculate

$$\begin{aligned} \int _{\varOmega }\left( a(x)-\tilde{a}\right) u_1(t,x) \mathrm {d}x = \int _{\varOmega _a}\left( a(x)-\tilde{a}\right) u_1(t,x) \mathrm {d}x + \int _{\varOmega {\setminus } \varOmega _a}\left( a(x)-\tilde{a}\right) u_1(t,x) \mathrm {d}x, \end{aligned}$$

where $\varOmega _a$ is defined in the expression (4). Consequently, using boundedness of $\rho _1$, boundedness of *a*(*x*) as well as Lemma 3, we obtain that

$$\begin{aligned} \int _{\varOmega }\left( a(x)-\tilde{a}\right) u_1(t,x) \mathrm {d}x \,\, { \mathop {\longrightarrow }\limits ^{t\rightarrow \infty }}\,\, 0, \end{aligned}$$

and hence we conclude that system (25) fulfills the assumptions of Lemma 7. Consequently, we obtain that the total mass of a solution of system (3) converges to a globally stable equilibrium, which is equal to the equilibrium of the ordinary differential equations model (20) corresponding to the maximum value of the self-renewal parameter ${\bar{a}}$. Thus, we have proven the assertion of Theorem 2.

## Proof of the convergence result

Finally, we obtain the main assertion.

### *Proof (of Theorem 1)*

Lemma 3 implies that the solutions of system (3) decay exponentially to zero in all points $x \notin \varOmega _a$. We consider two cases (compare Assumptions 1 (iv)):

(i) $$\varOmega _a= \{ {\bar{x}}\} $$:
  Convergence to a stationary solution follows from the convergence of mass given by Theorem 2. Hence, the solutions converge to measures concentrated at ${\bar{x}}$:
  $$\begin{aligned} u_i(t,\cdot )\mathcal L^1 \, \mathop {\longrightarrow }\limits ^{t\rightarrow \infty } c_i \delta _{{\bar{x}}}, \quad \text {for} \quad i=1,2, \end{aligned}$$
  where $\mathcal L^1$ denotes a one dimensional Lebesgue measure and $u_i(t,\cdot )\mathcal L^1$ is the measure which Radon–Nikodym derivative with respect to $\mathcal L^1$ is equal to *u*, $\delta _{{\bar{x}}}$ is a Dirac measure localised at ${\bar{x}}$ and $c_i$, $i=1,2$, are the stationary masses, i.e. $c_1=\bar{\rho }_1=\frac{d}{p}\frac{2{\bar{a}}-1}{K}$ and $c_2=\bar{\rho }_2=\frac{2{\bar{a}}-1}{K}$.
  The convergence result can be understood in a suitable metric on the space of positive Radon measures. We apply here the flat metric $\rho _F$, also known as the bounded Lipschitz distance (Neunzert 1981). For completeness of presentation, the definition and basic properties of this metric are provided in Appendix. To estimate the distance between a solution $u_i(t,\cdot )$ and the stationary measure $c_i \delta _{{\bar{x}}}$, $i=1,2$, we use the following inequality for the distance of two measures $\mu$ and $\nu$
  26 $$\begin{aligned} \rho _{F}(\mu ,\nu )\le \min \{\mu (\varOmega ),\nu (\varOmega )\} W_1\left( \frac{\mu }{\mu (\varOmega )},\frac{\nu }{\nu (\varOmega )}\right) + |\mu (\varOmega )-\nu (\varOmega )|. \end{aligned}$$
  For the proof of this inequality we refer to Carrillo et al. (2012) and Jabłoński and Marciniak-Czochra (2013). Here $W_1\left( \frac{\mu }{\mu (\varOmega )},\frac{\nu }{\nu (\varOmega )}\right)$ denotes the Wasserstein distance between two probabilistic measures; see Appendix for the definition of the Wasserstein metric.
  We calculate, for $i=1,2$,
  27 $$\begin{aligned} \rho _{F}\left( u_i(t,\cdot )\mathcal L^1,c_i \delta _{{\bar{x}}}\right) \le \min \{\rho _i,c_i\} W_1\left( \frac{u_i(t,\cdot )\mathcal L^1}{\rho _i},\delta _{{\bar{x}}} \right) + |\rho _i-c_i |. \end{aligned}$$
  The first term on the right hand-side of inequality (27) can be estimated using the exponential estimates of Lemma 2. To show that it converges to zero we apply the Kantorovich–Rubinstein Theorem (Villani 2003, 2006) and use the equivalent definition of the Wasserstein metric given as the cost of optimal transport with the cost function $|x - y|$, i.e.
  28 $$\begin{aligned} W_1\left( \frac{\mu }{\mu (\varOmega )}, \frac{\nu }{\nu (\varOmega )}\right) : = \inf _{\gamma \in {\mathcal P}(\varOmega )\times {\mathcal P}(\varOmega )} \int _{\varOmega \times \varOmega } |x - y|\; \gamma (\mathrm {d}x,\mathrm {d}y), \end{aligned}$$
  where $\gamma \in \varGamma \left( \frac{\mu }{\mu (\varOmega )}, \frac{\nu }{\nu (\varOmega )}\right)$ is a joint distribution (probabilistic measure) with the marginal distributions $\frac{\mu }{\mu (\varOmega )}$ and $\ \frac{\nu }{\nu (\varOmega )}$, and where
  $$\begin{aligned} \varGamma \left( \frac{\mu }{\mu (\varOmega )},\frac{\nu }{\nu (\varOmega )}\right) =&\Big \{ \gamma \in {\mathcal P}(\varOmega \times \varOmega )\gamma (B \times \varOmega ) = \frac{\mu (B)}{\mu (\varOmega )}, \\&\; \gamma (\varOmega \times B) = \frac{\nu (B)}{\nu (\varOmega )}, \; B \in {\mathcal B}(\varOmega ) \Big \}. \end{aligned}$$
  is the family of all joint distributions with marginal distributions $\frac{\mu }{\mu (\varOmega )}$ and $\ \frac{\nu }{\nu (\varOmega )}$.
  We estimate the difference between a normalised solution
  $$\begin{aligned} \pi _i(t):=\frac{u_i(t,\cdot )}{\rho _i(t)}\mathcal L^1 \end{aligned}$$
  and its limit $\delta _{{\bar{x}}}$, $i=1,2$. Using a joint distribution $\gamma _i = \delta _{{\bar{x}}} \otimes \pi _i$, $i=1,2$, we obtain
  29 $$\begin{aligned} W_1(\pi _i(t), \delta _{{\bar{x}}}) \le \int _{\varOmega } |{\bar{x}} - y|\; \pi _i(t) (\mathrm {d}y), \end{aligned}$$
  To show that the right-hand side of inequality (29) converges to zero, we define a set $\varOmega _{a-{\varepsilon }}=\left\{ x: a(x)>{\bar{a}} - \varepsilon \right\}$. For $\varepsilon$ small enough, there exists $\tilde{\varepsilon }>0$ such that the set $\varOmega _{a- \varepsilon }$ is contained in a $\tilde{\varepsilon }-$neighbourhood of $\varOmega _{a}$, i.e. $\varOmega _{a- \varepsilon } \in [{\bar{x}}- \tilde{\varepsilon }, {\bar{x}} + \tilde{\varepsilon }]$. By Lemma 2, $\pi _i(t)\left( \varOmega {\setminus } [{\bar{x}}- \tilde{\varepsilon }, {\bar{x}} + \tilde{\varepsilon }]\right) \rightarrow 0$ for $t\rightarrow \infty$. Therefore, we obtain
  $$\begin{aligned} W_1(\pi _i(t), \delta _{{\bar{x}}})\le & {} \int _{\varOmega {\setminus } [{\bar{x}}- \tilde{\varepsilon }, {\bar{x}} + \tilde{\varepsilon }]} |{\bar{x}} - y|\; \pi _i(t) (\mathrm {d}y) + \int _{[{\bar{x}}- \tilde{\varepsilon }, {\bar{x}} + \tilde{\varepsilon }]} |{\bar{x}} - y|\; \pi _i(t) (\mathrm {d}y) \\\le & {} \sup \limits _{x\in \varOmega } |{\bar{x}} - x| \pi _i(t)\left( \varOmega {\setminus } [{\bar{x}}- \tilde{\varepsilon }, {\bar{x}} + \tilde{\varepsilon }]\right) + \tilde{\varepsilon }\rightarrow \tilde{\varepsilon }, \quad \text {for} \quad t\rightarrow \infty . \end{aligned}$$
  Since the above convergence holds for any $\tilde{\varepsilon }>0$, we conclude that
  $$\begin{aligned} \lim \limits _{t \rightarrow \infty } W_1(\pi _i(t), \delta _{{\bar{x}}}) = 0. \end{aligned}$$
  Convergence of the second term in formula (27) is due to Theorem 2. Hence, we obtain that
  $$\begin{aligned} \lim \limits _{t \rightarrow \infty } \rho _{F}\left( u_i(\cdot ,t)\mathcal L^1,c_i \delta _{{\bar{x}}}\right) =0. \end{aligned}$$
(ii) $$\mathcal L^1 (\varOmega _a)>0$$:
  If $\varOmega _a$ is a set with positive measure, no singularities emerge due to the uniform boundedness of the total mass. In this case, the solution tends to zero outside $\varOmega _a$ and to a positive $L^1$-function on $\varOmega _a$. Following Corollary 1, we conclude that the exact shape of the limit solution depends on the initial distribution.
(iii) If ${\bar{a}} = \max \nolimits _{x\in \overline{\varOmega }} a(x) \le \frac{1}{2}$, then the solutions converge exponentially to zero, what is a consequence of Eq. (3). We estimate
  $$\begin{aligned} \frac{d}{dt}\rho _1(t)\le \left( \frac{1}{1+K \rho _2(t)}-1\right) p\rho _1(t) \le -C \rho _1(t), \end{aligned}$$
  where $C= - \left( \frac{1}{1+K \min _{t\in [0,\infty )}\rho _2(t)}-1\right) p >0$, due to Lemma 1. Hence, using the Gronwall inequality, we obtain the exponential decay to zero. Finally, convergence $\rho _2(t) \rightarrow 0$ as $t\rightarrow \infty$ follows from the estimate
  $$\begin{aligned} \frac{d}{dt}\rho _2(t)\le 2p \rho _1(t) -d \rho _2(t). \end{aligned}$$
  Since the solutions $(u_1,u_2)$ are nonnegative, they converge to zero in $L^1(\varOmega )$.

Finally, we analyse the case with $\varOmega _a$ consisting of two points and prove the co-existence and the extinction result.

### *Proof (of Theorem 3)*

(i) We investigate dynamics of the mass of a solution of system (3) around the points of $\varOmega _a$. Let us assume that there exists a diffeomorphism $\varPhi \in C^1(U_1)$, where $U_1$ is an open neighbourhood of ${\bar{x}}_1$, such that $\varPhi ({\bar{x}}_1)={\bar{x}}_2$ and $a(x)=a(\varPhi (x))$ for all $x\in U_1$. Using the explicit form of the solution (13) and the property $\varPhi ({\bar{x}}_1)={\bar{x}}_2$, we obtain

30 $$\begin{aligned} \int \limits _{U_1} u_1(t,x) \mathrm {d}x = \int \limits _{U_1} u_1(t,\varPhi (x)) \frac{u^0_1(x)}{u^0_1(\varPhi (x))} \mathrm {d}x, \end{aligned}$$

Changing variables on the right hand-side of (30) leads to

31 $$\begin{aligned} \int \limits _{U_1} u_1(t,x) \mathrm {d}x = \int \limits _{\varPhi (U_1)} u_1(t,y) \frac{u^0_1(\varPhi ^{-1}(y))}{u^0_1(y)} J\varPhi ^{-1}(y) \mathrm {d}y, \end{aligned}$$

where $J\varPhi$ is Jacobian of the diffeomorphism $\varPhi$.

Since $\frac{u^0_1(\varPhi ^{-1}(y))}{u^0_1(y)} J\varPhi ^{-1}(y)$ does not depend on time and is continuous with respect to *y* and since *u*(*t*, *x*) converges pointwise to zero outside $\varOmega _a=\{{\bar{x}}_1, {\bar{x}}_2\}$ (see Lemma 3), we obtain

32 $$\begin{aligned} \lim \limits _{t\rightarrow +\infty } \int \limits _{U_1} u_1(t,x) \mathrm {d}x = \frac{u^0_1({\bar{x}}_1)}{u^0_1({\bar{x}}_2)} J\varPhi ^{-1}({\bar{x}}_2) \lim \limits _{t\rightarrow +\infty } \int \limits _{\varPhi (U_1)} u_1(t,y) \mathrm {d}y, \end{aligned}$$

Hence, the solution converges to a measure $c_{1,1}\delta _{{\bar{x}}_1} + c_{1,2}\delta _{{\bar{x}}_2}$ with strictly positive $c_{1,1}$ and $c_{1,2}$ such that

33 $$\begin{aligned} \frac{c_{1,1}}{c_{1,2}}=\frac{u^0_1({\bar{x}}_1)}{u^0_1({\bar{x}}_2)} J\varPhi ^{-1}({\bar{x}}_2). \end{aligned}$$

Since the total mass of $u_1$ is equal to $c_{1,1}+c_{1,2}=\bar{\rho }_1$, where $\bar{\rho }_1$ is given in Corollary 2, the constants $c_{1,1}$ and $c_{1,2}$ are uniquely determined. Relationship (33) indicates that the mass distribution between the different concentration points depends on the shape of the function *a*(*x*) and on the initial data.

(ii) Now, we consider the case where the mapping $\varPhi$ defined above is only a homeomorphism and $J\varPhi ^{-1}$ is continuous but $J\varPhi ^{-1}({\bar{x}}_2)=0$. Hence, Eq. (32) yields that $\lim \nolimits _{t\rightarrow +\infty } \int \nolimits _{U_1} u_1(t,x) \mathrm {d}x =0$, which implies that the solution converges to a mass $c_{1,2}\delta _{{\bar{x}}_2}$ with $c_{1,2}=\bar{\rho }_1$.

### *Remark 5*

Continuity of $\frac{u^0_1(\varPhi ^{-1}(y))}{u^0_1(y)} J\varPhi ^{-1}(y)$ requires continuity of the initial data and strict positivity of $u^0_1$ on $\varOmega _a$, which is reflected in the stronger assumptions of the theorem compared to Assumption 1.

## Extension to initial data in the space of Radon measures

The phenomenon of mass concentration provides a motivation to consider the model in the space of positive Radon measures, as defined by the following equations

34 $$\begin{aligned} \frac{d}{d t} \mu _1(t)(B)= & {} \int \limits _{B}\left( \frac{2a(x)}{1+K\rho _2(t)}-1\right) p \mu _1(t)(\mathrm {d}x),\nonumber \\ \frac{d}{d t} \mu _2(t)(B)= & {} \int \limits _{B}2\left( 1-\frac{a(x)}{1+K\rho _2(t)}\right) p \mu _1(t) (\mathrm {d}x) - d\int \limits _{B} \mu _2(t)(\mathrm {d}x), \end{aligned}$$

with

35 $$\begin{aligned} \rho _i(t) = \int \limits _{\varOmega } \mu _i(t)(\mathrm {d}x), \quad i=1,2, \end{aligned}$$

with the initial data

36 $$\begin{aligned} \mu _1(0)= & {} \mu _1^0, \nonumber \\ \mu _2(0)= & {} \mu _2^0, \end{aligned}$$

where $\mu _i^0$ are nonnegative Radon measures for $i=1,2$. $x\in \varOmega \subset \mathbb {R}^n$, for some $n\ge 1$, denotes the state of a cell and, for every Borel subset $B \subset \varOmega$, $\mu _i(t)(B)=\int _{B} d\mu _i(t)$, $i=1,2$, are measures of cells in any of the states $x \in B$ at time *t*. Variable $\rho _i$ denotes the mass of all cells from the *i*th compartment. Measures $\mu (t)$ are $C^1$ functions of time with values in the space of positive Radon measures with the total variation norm. Therefore, the time derivatives in equations (34) are understood as derivatives of the functions with values in a Banach space.

Selection-mutation models in the spaces of positive Radon measures have been studied by many authors Ackleh et al. (1999), Ackleh et al. (2005), Bürger and Bomze (1996), Bürger (2000), Caizo et al. (2013), Cleveland and Ackleh (2013), Desvillettes et al. (2008). In this context, convergence of the solutions with respect to the Prokhorov metric has been considered in Ackleh et al. (1999). For the relation between the Prokhorov metric and the Wasserstein distance used in our paper we refer to Gibbs and Su (2017).

Steps of the proof of Theorem 1 can be repeated for the measure-valued solutions with some modifications of the lemmas which rely on point-wise estimates of the quotients of solutions. Assuming that the initial data are measures such that $\mu _1^0$ is absolutely continuous with respect to $\mu _2^0$, Lemma 4 can be reformulated for the model (34)–(36) by considering a Radon–Nikodym derivative

37 $$\begin{aligned} \left( D_{\mu _2(t)}\mu _1(t)\right) (x) = \lim _{r \rightarrow 0^+}\frac{\mu _1(t)(B_{x,r})}{\mu _2(t)(B_{x,r})} \end{aligned}$$

instead of the point-wise quotients.

Next technical difficulty appears in Lemma 2. To show the asymptotic behaviour of the measure-valued solutions, we can apply the framework developed in Bürger and Bomze (1996). In the remainder of this section, we briefly discuss this extension.

The first equation of the model (34)–(36) can be re-defined in the terms of a probabilistic measure modelling the frequency of a certain phenotype $x\in B$ in the population of mitotic cells $\mu _1$. It is given by the quotient

$$\begin{aligned} \pi (t)(B)={\frac{\mu _1(t)(B)}{\mu _1(t)(\varOmega )}}, \end{aligned}$$

where $B \subset \varOmega$ is a Borel set, as defined before.

Using the equation for $\mu _1$, we obtain

38 $$\begin{aligned} \frac{d}{d t}\pi (t)(B)=\frac{2p}{1+\rho _2(t)}\int \limits _{B}\left( a(x)-\int _{\varOmega } a(\xi )\; \pi (t)(\mathrm {d}\xi ) \right) \; \pi (t)(\mathrm {d}x). \end{aligned}$$

The model can be then formulated in the framework presented in the book by Bürger (Bürger 2000). Denoting the mean fitness by

39 $$\begin{aligned} \overline{\mathcal A}(t) = \frac{2p}{1+\rho _2(t)} \int _{\varOmega } a(\xi )\; \pi (t)(\mathrm {d}\xi ) \end{aligned}$$

and the multiplication operator $\mathcal A(t)$ by

40 $$\begin{aligned} \left( \mathcal A(t)\pi (t)\right) (B) =\frac{2p}{1+\rho _2(t)}\int \limits _{B}a(x) \pi (t)(\mathrm {d}x), \end{aligned}$$

we rewrite Eq. (38) as an ordinary differential equation in the space of Radon measures

41 $$\begin{aligned} \frac{d}{dt}\pi (t) = \mathcal A(t) \pi (t) - \overline{\mathcal A}(t) \pi (t). \end{aligned}$$

However, the obtained equation is more general than the abstract equation in Bürger (2000), due to the dependence of $\mathcal A$ on time. Nevertheless, it holds

$$\begin{aligned} \overline{\mathcal A}(t)=\left( \mathcal A(t) \pi (t)\right) (\varOmega ). \end{aligned}$$

Using the form of the operator (40), we rewrite it as a function of time $\alpha (t)=\frac{2p}{1+\rho _2(t)}$ multiplied by a time independent operator $\left( A \pi (t)\right) (B)=\int \nolimits _{B}a(x)\pi (t)(\mathrm {d}x)$,

42 $$\begin{aligned} \mathcal A(t) =\alpha (t) A. \end{aligned}$$

This structure allows to follow the lines of Bürger and Bomze (1996) and focus on a differential equation given by

43 $$\begin{aligned} \frac{d}{dt}Q(t)=\mathcal A(t)Q(t). \end{aligned}$$

The structure assures that the family of operators ${\mathcal A}$ commutes. The operator ${\mathcal A}$ is bounded and it generates a positive semigroup on the space of positive Radon measures ${\mathcal M^+}({\varOmega })$.

Since $\alpha$ is a strictly positive and bounded function, due to the properties of $\rho _2$ shown in Lemma 1, we can rescale time, $s=\int _0^t \alpha (\xi )\mathrm {d}\xi$, and obtain a linear autonomous differential equation

44 $$\begin{aligned} \frac{d}{ds}Q(s)= AQ(s). \end{aligned}$$

Equivalence to a linear differential equation yields convergence of solutions to a solution $\pi (t)$ with the support concentrated on the set of maximal value of *a*(*x*), ${\bar{a}}= \sup \nolimits _{x\in \varOmega \cap {\mathrm{supp}}(\mu _1^0)}a(x)$. The latter result is the extension of our Lemma 2 to the measure-valued solutions.

In summary, by adapting the framework developed in Bürger and Bomze (1996), our results can be extended to the measure-valued solutions in the case of the model of the clonal evolution without mutations. Asymptotic analysis carried out in Bürger and Bomze (1996) is based on the application of the infinite-dimensional version of the Perron-Frobenius Theorem, which is possible in models with dynamics governed by an irreducible operator. The latter is the case in models involving mutations described by an integral operator satisfying irreducibility conditions. That approach cannot be, however, directly applied to the extension of our model to the case with mutations. The difficulty is related to the estimates for the time dependent operator $\mathcal A$ defined in expression (40), which rely on the equations for the ratios of solutions in Lemma 4, or Radon–Nikodym derivatives (37), which cannot be established in the model with an additional nonlocal mutation operator. Therefore, including mutations in our model requires a different proof of the uniform boundedness and strict positivity of $\rho _2$ and extension of the analysis to the model with mutations remains an open question.

## Discussion

In this paper, a discrete multi-compartmental model of multiple cell lineages has been extended to a model coupling a two-stage differentiation structure with a continuous structure of phenotypes. The latter allows to investigate the role of the intra-cancer heterogeneity, including competition between healthy and cancer cells and dynamics of the multi-clonal structure of the system.

Based on recent analyses of the clones consisting of mutational variants in cancer (Miller et al. 2014), it follows that the dynamics of clone distributions may in many cases consist solely of change in relative frequencies of different clones. More specifically, the clones that have been dominant in the primary tumour, are out-competed by other clones in the relapsing or metastatic tumours, which had low frequencies in the primary. The model in this paper provides a “mechanistic” explanation for these observations, which is also mathematically rigorous.

Asymptotic analysis of the proposed system of integro-differential equations suggests that the selection process may be governed by the cell’s property of self-renewal that determines the fitness of each clone and ultimately leads to survival or extinction.

Theorem 1 shows that, in a well-mixed cell production system, a negative nonlinear feedback such as that the one proposed in Lander (2009), Lander et al. (2009), Marciniak-Czochra et al. (2009), leads to the selection of the subpopulation with the superior self-renewal potential. The assumption that the cell population is well-mixed leads to the nonlocal effect and is modelled using the integral term. This assumption reflects well the structure of the hematopoietic system. Consequently, our results suggest that the greater clonal heterogeneity observed in solid cancers than in blood cancers may be due to spatial effects of the cell-to-cell interactions. Additionally, Theorem 3 suggests some explanation of the co-existence of different clones having the same fitness.

The results stress the importance of self-renewal in cancer dynamics and allow concluding that slowly proliferating cancer cells with a high self-renewal potential are able to outcompete the cells that divide faster. It suggests an explanation of the clinical dynamics such as resistance to treatment. Importance of this observation in the context of the leukemia evolution, the response to chemotherapy and the dynamics of the disease relapses has been discussed in Stiehl et al. (2014). The results obtained provide an explanation of the observed clonal selection in the acute myeloid leukemia in the course of the disease development and the relapse after chemotherapy reported by Ding et al. (2012). Recently, fitting the AML model to patients’ data has suggested that an increased self-renewal is correlated with a poor patient prognosis (Stiehl et al. 2015).

## Appendix

### Flat metric

We present here basic results concerning the space of positive Radon measures equipped with the flat metric $\rho _F$, known also as the bounded Lipschitz distance (Neunzert 1981).

#### **Definition 1**

Let $\mu , \nu \in {\mathcal M^+}({\varOmega })$. The distance function $\rho _F : {\mathcal M^+}({\varOmega }) \times {\mathcal M^+}({\varOmega }) \rightarrow [0, \infty )$ is defined by

45 $$\begin{aligned} \rho _F(\mu , \nu ) := \displaystyle \sup \left\{ \int _{\varOmega } \psi d (\mu - \nu ) \big | \, \psi \in C^1({\varOmega }), \Vert \psi \Vert _{W^{1,\infty }} \le 1 \right\} , \end{aligned}$$

where

$$\begin{aligned} \Vert \psi \Vert _{W^{1,\infty }} := \max \{\Vert \psi \Vert _{\infty },\Vert \partial _x \psi \Vert _{\infty }\}. \end{aligned}$$

The $\rho _F$ distance metrizes both weak* and narrow topologies on each tight subset of Radon measures with uniformly bounded total variation (Schwartz 1973; Ambrosio et al. 2000).

#### *Remark 6*

Every bounded Radon measure on a bounded set $\varOmega$ has an integrable first moment and hence the distance $\rho _F$ is finite.

#### **Proposition 1**

Flat metric satisfies the following properties:

- scale-invariance
  $$\begin{aligned} \rho _F(\theta \cdot \mu ,\theta \cdot \nu )= \theta \rho _F(\mu ,\nu ).\\ \end{aligned}$$
- translation-invariance
  $$\begin{aligned} \rho _F(T_{x}\mu ,T_{x}\nu )= \rho _F(\mu ,\nu ). \end{aligned}$$

Completeness of the space $\left( \mathcal {M}^+(\varOmega ), \rho _F\right)$ is the result of $\left( \mathcal {M}^+(\varOmega ),\rho _F\right)$ being a subspace of $\left( W^{1,\infty }(\varOmega )\right) ^*$ and the equivalence of the flat metric convergence and weak* convergence in $\mathcal {M}^+(\varOmega )$, which is complete with respect to weak* convergence. Inclusion $\left( \mathcal {M}^+(\varOmega ),\rho _F\right) \subset \left( W^{1,\infty }(\varOmega )\right) ^*$ is proven using a standard approximation argument for the test functions and Proposition 1.

$$\begin{aligned} \rho _F(\mu ,\nu )= & {} \sup \left\{ \int \limits _{\varOmega } \psi \,\mathrm {d}(\mu -\nu )\left| \psi \in C^1(\varOmega ), \left\| \psi \right\| _{W^{1, \infty }(\varOmega )} \le 1 \right. \right\} \\= & {} { \sup \left\{ \frac{1}{\theta }\int \limits _{\varOmega } \varphi \, \mathrm {d}(\mu -\nu )\left| \varphi \in W^{1,\infty }(\varOmega ), \left\| \varphi \right\| _{W^{1,\infty }(\varOmega )} \le \theta \right. \right\} } \\= & {} \left\| \mu -\nu \right\| _{\left( W^{1,\infty }(\varOmega )\right) ^*} \end{aligned}$$

Thus the flat metric is the metric induced by the dual norm of $W^{1,\infty }(\varOmega )$; see e.g. Gwiazda et al. (2010), Gwiazda and Marciniak-Czochra (2010), Müller and Ortiz (2004), Zhidkov (1998).

### Wasserstein metric

The Wasserstein metric $W_1: {\mathcal P}(\varOmega )\times {\mathcal P}(\varOmega ) \longrightarrow [0,\infty )$ in its dual representation is defined by

$$\begin{aligned} \begin{array}{@{}rcl@{}l@{}l@{}l@{}l@{}} W_1\left( \frac{\mu }{\mu (\varOmega )},\frac{\nu }{\nu (\varOmega )} \right):= & {} \displaystyle \sup&\Bigg \{&\displaystyle \int _{\varOmega } \; \psi \,\, d \left( \frac{\mu }{\mu (\varOmega )} - \frac{\nu }{\nu (\varOmega )}\right) \;&\Big |\; \psi \in C^1(\varOmega ), \;\, {\mathrm{Lip}} \, \psi \le 1&\Bigg \}. \end{array} \end{aligned}$$

For more information on the Wasserstein metric we refer to Villani (2006), Villani (2003).
